# Assessment of comorbidities and prognosis in patients with COPD diagnosed with the fixed ratio and the lower limit of normal: a systematic review and meta-analysis

**DOI:** 10.1186/s12931-020-01450-9

**Published:** 2020-07-16

**Authors:** Huaiyu Xiong, Qiangru Huang, Tiankui Shuai, Lei Zhu, Chuchu Zhang, Meng Zhang, Yalei Wang, Jian Liu

**Affiliations:** 1 The First Clinical Medical College of the First Hospital of Lanzhou University, Lanzhou, 730000 China; 2 Department of Intensive Care Unit, The First Hospital of Lanzhou University, No.199 Donggang West Road, Chengguan District, Lanzhou City, 730000 Gansu Province China; 3 Evidence-Based Medicine Center, School of Basic Medical Sciences, Lanzhou University, Lanzhou, 730000 China

**Keywords:** COPD, Fixed-ratio, Lower limit of normal, Comorbidity

## Abstract

**Background:**

Currently, the diagnosis of chronic obstructive pulmonary disease (COPD) is not uniform, COPD guidelines recommend fixed ratio (FR), whereas ATS and ERS define airflow obstruction based on lower limit of normal (LLN). We aim to determine if there is difference between the two diagnostic criteria for morbidity, mortality, exacerbation.

**Methods:**

Four databases and all relevant studies from the references were searched from inception to June 25, 2019, to find studies that described the rate of comorbidity, the exacerbation rates, mortality in COPD patients. Data analysis was performed using STATA/SE 14.0 and followed the standard of Cochrane Collaboration. A sensitivity analysis was performed to find the source of heterogeneity.

**Results:**

Thirteen studies and 154,447 participants were finally included in this meta-analysis. The 11 cohort studies and 2 cross-sectional studies were all high-quality. Patients with airflow limitation according to either FR or LLN had higher mortality (HR_FR+/LLN-_ = 1.27, 95% CI = 1.14–1.42; HR_FR−/LLN+_ = 1.83, 95% CI = 1.17–2.86) than those who met neither criteria. When compared with the FR−/LLN- criteria, those who met the FR criteria were more likely to exacerbate (HR _FR+/LLN-_ = 1.64, 95% CI = 1.09–2.46; HR _FR−/LLN+_ = 1.58, 95% CI = 0.70–3.55). The meta-analysis for comorbidities showed no significant difference between patients who met neither criteria and those who met LLN or FR criteria.

**Conclusion:**

The patients with airflow limitations according to FR were more likely to exacerbate than those with LLN only. Patients that met either FR or LLN were more likely to have higher mortality than FR−/LLN-. There was no difference between the FR+/LLN- and FR−/LLN+ groups for the occurrence of comorbidities.

## Background

Chronic obstructive pulmonary disease (COPD) is a non-curable disease with high morbidity and mortality [1]. Globally, it is the 3rd leading cause of death [2]. In America in the 2000s, the prevalence of COPD was 24 million, and half of these were undiagnosed [3]. In 2015, 99.9 million Chinese were diagnosed with COPD, which accounted for 8.6% of the total population [4]. Patients with COPD always have respiratory symptoms and often have comorbidities [5]. Moreover, several co-morbidities of COPD, such as cardiovascular disease and diabetes mellitus have been reported to exacerbate the mortality of COPD [6, 7].

COPD guidelines recommend the use of post-bronchodilator forced expiratory volume in 1 s (FEV_1_)/ forced vital capacity (FVC) ratio to diagnose patients with chronic respiratory symptoms or those at risk [1, 8, 9]. However, the American Thoracic Society (ATS) and European Respiratory Society (ERS) recommend the use of a threshold below the lower limit of normal (LLN) adjusted for age criteria to establish a diagnosis of COPD [10]. Given these complex diagnostic methods, the current data about lung function and prognosis of COPD are difficult to compare [11]. Several studies have demonstrated that a decline of lung function is a marker for premature death, especially in cardiovascular disease [12, 13]. Additionally, the criteria of FEV1/FVC<0.70 underestimates the prevalence of COPD, especially among people under 45 years of age [14, 15]. Colak Y et al. reported that underdiagnosis of COPD is closely related to poor prognosis, even among asymptomatic individuals [16]. A study demonstrated that the LLN criterion does not identify important pulmonary pathologies and respiratory-related complications in a large number of patients with COPD compared to a fixed ratio (FR) [17].

Identifying alternate prognoses and outcomes for COPD patients (that meet a different set of diagnostic criteria) can lay the groundwork for determining which diagnostic criterion is better. A meta-analysis comparing the risk of comorbidities and mortality in patients with different diagnostic criteria has not been performed to date. Additionally, high-quality meta-analysis is increasingly recognized as a key factor in obtaining a high level of evidence. Therefore, we conducted a meta-analysis to determine if there is a difference between the 2 diagnostic criteria for morbidity, mortality, and exacerbation.

## Research design and methods

The methods used in this study followed guidelines from the Cochrane Handbook for Systematic Reviews of Interventions. The meta-analysis was conducted according to the Preferred Reporting Items for Systematic Reviews and Meta-analysis (PRISMA) guidelines [18] and presented based on Assessing the Methodological Quality of Systematic Reviews (AMSTAR) guidelines.

### Data sources and searches

We searched the PubMed/Medline, Cochrane Library, Web of Science, and Embase databases from inception to June 25, 2019, to identify studies that captured data on the rate of comorbidity and mortality in COPD patients. All relevant studies from the references were screened to complement these databases. There was no language restriction during our search process. The search strategy according to MESH terms and keywords in PubMed were: (“Pulmonary Disease, Chronic Obstructive”[Mesh]) OR (COPD OR Chronic Obstructive Pulmonary Disease) OR (“Lung Diseases, Obstructive”[Mesh]) OR (Lung Disease, Obstructive OR Obstructive Lung Disease) OR (Bronchitis, Chronic OR Chronic Bronchitis) OR “Pulmonary Emphysema”[Mesh]) OR (Emphysema, Pulmonary OR Focal Emphysema) AND (fixed ratio OR fixed-ratio OR FEV1/FVC0.7 OR LLN OR Lower limit of normal). The detailed search strategy is given in Appendix S1.

The inclusion criteria for the studies were: 1. Observational studies or Random Controlled Trials; 2. Studies that included adult patients; 3. Studies that described different disease progression and outcomes from different diagnostic criteria (both FR and LLN).

Exclusion criteria: 1. Participants with pre-existing asthma and lung cancer; 2. Participants with lung injury; 3. Case reports, animal and cell studies, reviews and meta-analysis, and conference abstracts; 4. Studies that included children.

We defined “FR+/LLN+” as individuals with FEV1/FVC **<** 0.7 and FEV1/FVC < LLN, “FR+/LLN-” as individuals with FEV1/FVC **<** 0.7 but FEV_1_/FVC > LLN, “FR−/LLN-” as individuals with FEV1/FVC ≥ 0.7 and FEV1/FVC > LLN, “FR−/LLN+” as individuals with FEV1/FVC ≥ 0.7 but FEV1/FVC **<** LLN.

### Data extraction and quality assessment

Two researchers (X.H.Y. and H.Q.R.) extracted baseline characteristics independently, which included: name of the first author, publication year, study design, country, study samples, age, number of male participants, smoking status, hazards ratio (HR) for mortality and exacerbation, odds ratio (OR) for comorbidities. Any disagreements were resolved by discussion between the 2 authors.

### Data analysis

The data were analyzed by STATA/SE 14.0. HR and 95% Confidence Interval (CI) were estimated for mortality and exacerbation of COPD. OR and 95% CI were also calculated for the comorbidities of COPD patients. The ORs were calculated by Review Manager (Revman 5.1) when it was not mentioned in the original studies. Heterogeneity was assessed using the *I*^2^ statistic and *P*-value. The pooled OR or HR was analyzed by a random-effects model if the heterogeneity was high; otherwise, a fixed-effects model was used. A sensitivity analysis was performed by removing the studies one by one. If there were > 10 publications, a funnel plot and an Egger’s test was used to assess the publication bias [19]. A *P*-value <0.05 was considered as statistically significant.

Two researchers (X.H.Y and H.Q.R) assessed the quality of the included studies independently. We used the Newcastle Ottawa Scale (NOS), which consisted of selection, comparability, and outcome assessment, to assess the quality of the included cohort studies. A study was considered to have a low risk of bias when it scored > 7 out of 9; moderate risk was defined as 5–7, and high risk was defined as < 5. The quality of the cross-sectional studies was assessed by the Agency for Healthcare Research and Quality (AHRQ) scale, which contains 11 terms. Studies that scored 0–3, 4–7, and 8–11 were considered as low, moderate, and high quality respectively. The Engauge Digitizer (version 4.1) graphical data extraction software was used to extract data that were only provided by images.

## Results

### Study retrieved and characteristics

**Fig. 1:**
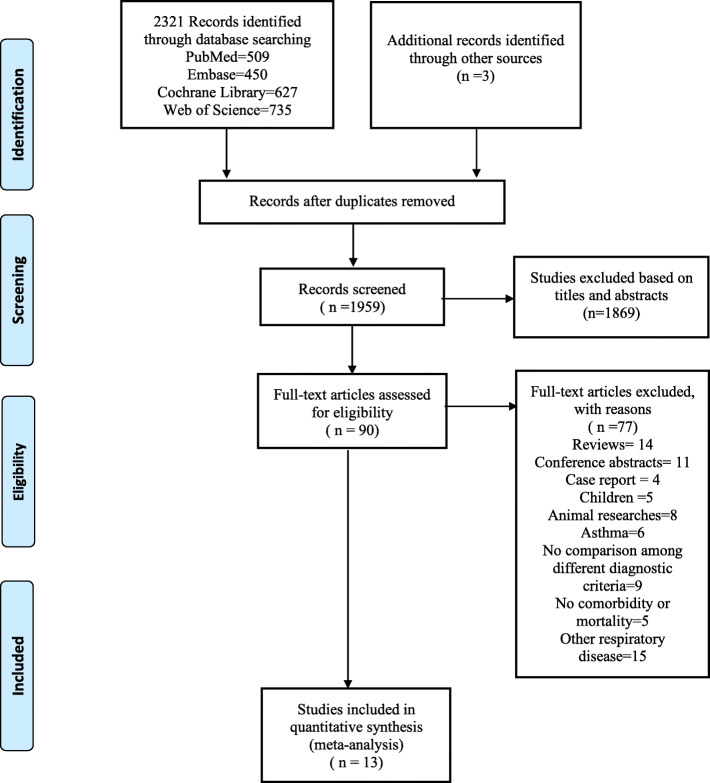
PRISMA (preferred reporting items for systematic reviews and meta-analyses) flow diagram and exclusion criteria

The meta-analysis retrieved 2321 studies and an additional 3 after searching across 4 databases and references. After removing duplicates, 1959 studies were screened for potential eligibility based on the title and abstract. Two researchers (S.T.K and Z.L) independently reviewed the full text of 90 potentially eligible studies. Finally, 13 studies [17, 20–31] and 154,447 participants were included in the meta-analysis (Fig. 1).

Of the final 13 studies, 11 were cohort studies and 2 were cross-sectional studies. According to the quality assessment, 11 cohort studies were rated high; 6 studies [17, 21, 22, 24, 29, 31] scored 9/9 stars, 3 [25, 26, 30] scored 8/9, and 2 [20, 27] scored 7/9 according to the NOS criteria. The 2 cross-sectional studies scored high on the methodological quality evaluation with one [28] scoring 8 and the other [23] scoring 10 on the AHRQ scale (Supplementary S1, S2).

The characteristics of the included studies are shown in Table 1. The sample sizes of the included studies ranged from 689 to 95,288, ages ranged from 20 to 100, the proportion of males varied from 23.1 to 100%, and a positive history of smoking varied from 47.2 to 84.5%.

**Table 1:** Characteristics of included studies (*n* = 13)

Study	Year	Country	Study Design	N	Age (yrs.)	Male (%)	History of smoking (%)	Comorbidities	NOS	AHRQ
Lea Sator	2019	Austria	Cohort	16,177	≥40	7894 (48.8)	47.2	①③④	7	NA
Yunus Çolak	2018	Denmark	Cohort	95,288	20–100	42,883 (45.0)	57.3	①②④	9	NA
Martin R Miller	2018	England	Cross-sectional	3721	40–79	1965 (52.8)	54.6	①②③④	NA	10
Claudio Pedone	2017	Italy	Cohort	882	≥65	203 (23.1)	NA	①②③④	9	NA
Suneela Zaigham	2015	Sweden	Cohort	689	≥55	689 (100)	84.5	④	9	NA
Wouter van Dijk	2015	Canada	Cross-sectional	4882	57.0 ± 11.0	2093 (43)	57	①	NA	8
Eralda Turkeshi	2015	Belgium	Cohort	411	84.6 ± 3.4	152 (37)	NA	NA	9	NA
Surya P Bhatt	2014	USA	Cohort	7743	45–80	4342 (56.1)	NA	NA	9	NA
Per Wollmer	2013	Sweden	Cohort	689	≥55	689 (100)	84.5	①	8	NA
Firdaus A	2013	Netherlands	Cohort	1108	62.5 ± 5.2	NA	52.8	NA	7	NA
David M Mannino	2006	USA	Cohort	4965	≥65	2155 (43.4)	54.1	NA	8	NA
David M. Mannino	2012	USA	Cohort	13,847	≥25	6495 (46.9)	56.3	NA	8	NA
Reinier P. Akkermans	2012	Netherlands	Cohort	4045	35–60	2513 (62.1)	NA	NA	9	NA

*Abbreviations*: *NOS* Newcastle Ottawa Scale, *AHRQ* agency for healthcare research and quality; Comorbidities: ① heart disease, ② heart failure, ③ stroke, ④ Diabetes Mellitus; *NA* not applicable

### Mortality and exacerbation

**Fig. 2:**
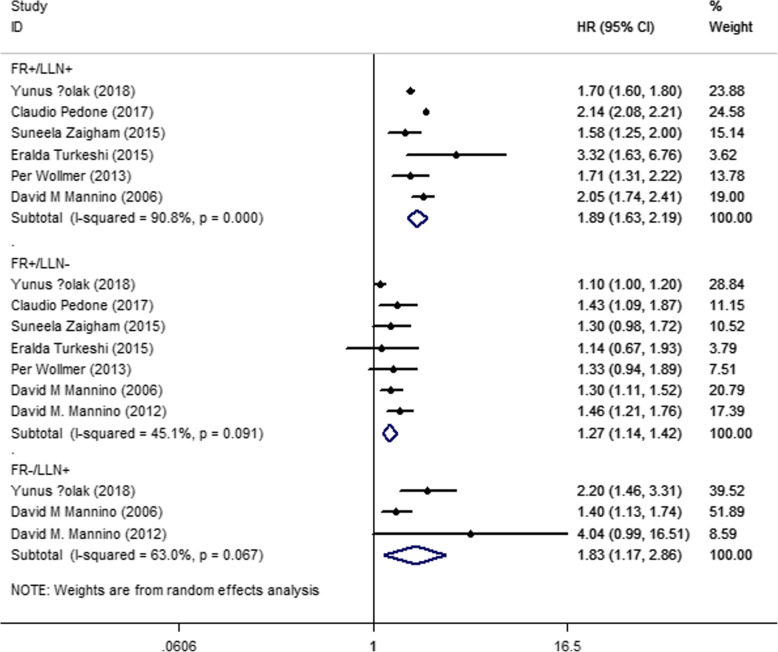
Plot of mortality for different diagnostic criteria, compared with those met neither (FR+/LLN+: FEV1/FEV < 0.7 and FEV1/FVC < LLN, FR+/LLN-: FEV1/FEV < 0.7 but FEV1/FVC > LLN, FR−/LLN+: FEV1/FEV ≥ 0.7 but FEV1/FVC < LLN)

**Fig. 3:**
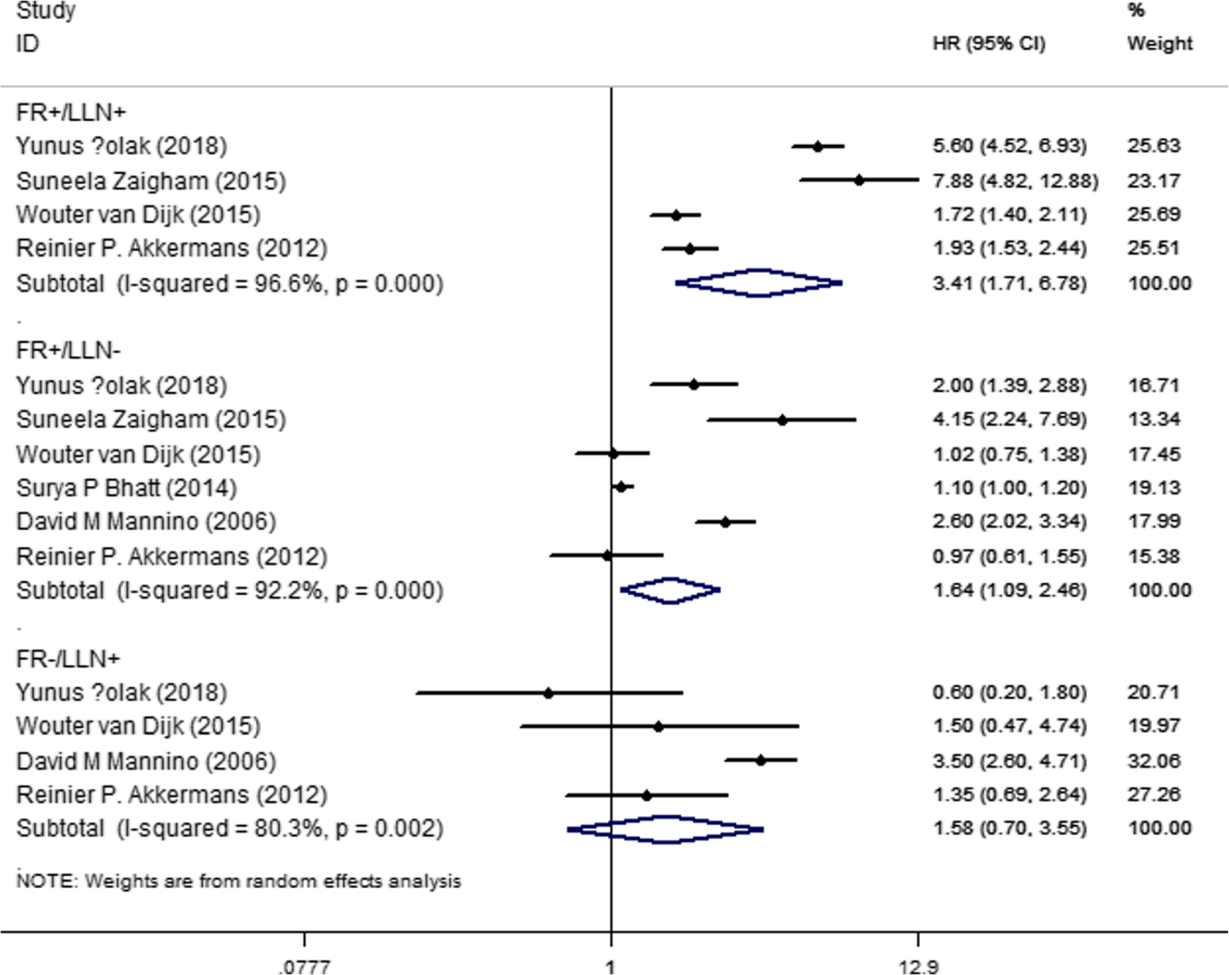
Forest plot of exacerbation for different diagnostic criteria, compared with those met neither (FR+/LLN+: FEV1/FEV < 0.7 and FEV1/FVC < LLN, FR+/LLN-: FEV1/FEV < 0.7 but FEV1/FVC > LLN, FR−/LLN+: FEV1/FEV ≥ 0.7 but FEV1/FVC < LLN)

Seven studies reported mortality rates. The participants with airflow limitation according to either FR or LLN were more likely to die (HR_FR+/LLN+_ = 1.89, 95% CI = 1.63–2.19; HR_FR+/LLN-_ = 1.27, 95% CI = 1.14–1.42; HR_FR−/LLN+_ = 1.83, 95% CI = 1.17–2.86) than those who met neither criteria (Fig. 2). Five studies reported exacerbation rates. The symptoms of the patients who met both criteria or only FR were more likely to exacerbate; only those who met the LLN criteria had no significant difference when compared to those who were TN (HR _FR+/LLN+_ = 3.41, 95% CI = 1.71–6.78; HR _FR+/LLN-_ = 1.64, 95% CI = 1.09–2.46; HR _FR−/LLN+_ = 1.58, 95% CI = 0.70–3.55) (Fig. 3). Although a sensitivity analysis was performed to exclude studies one by one, the results remained unchanged.

### Comorbidity

**Fig. 4:**
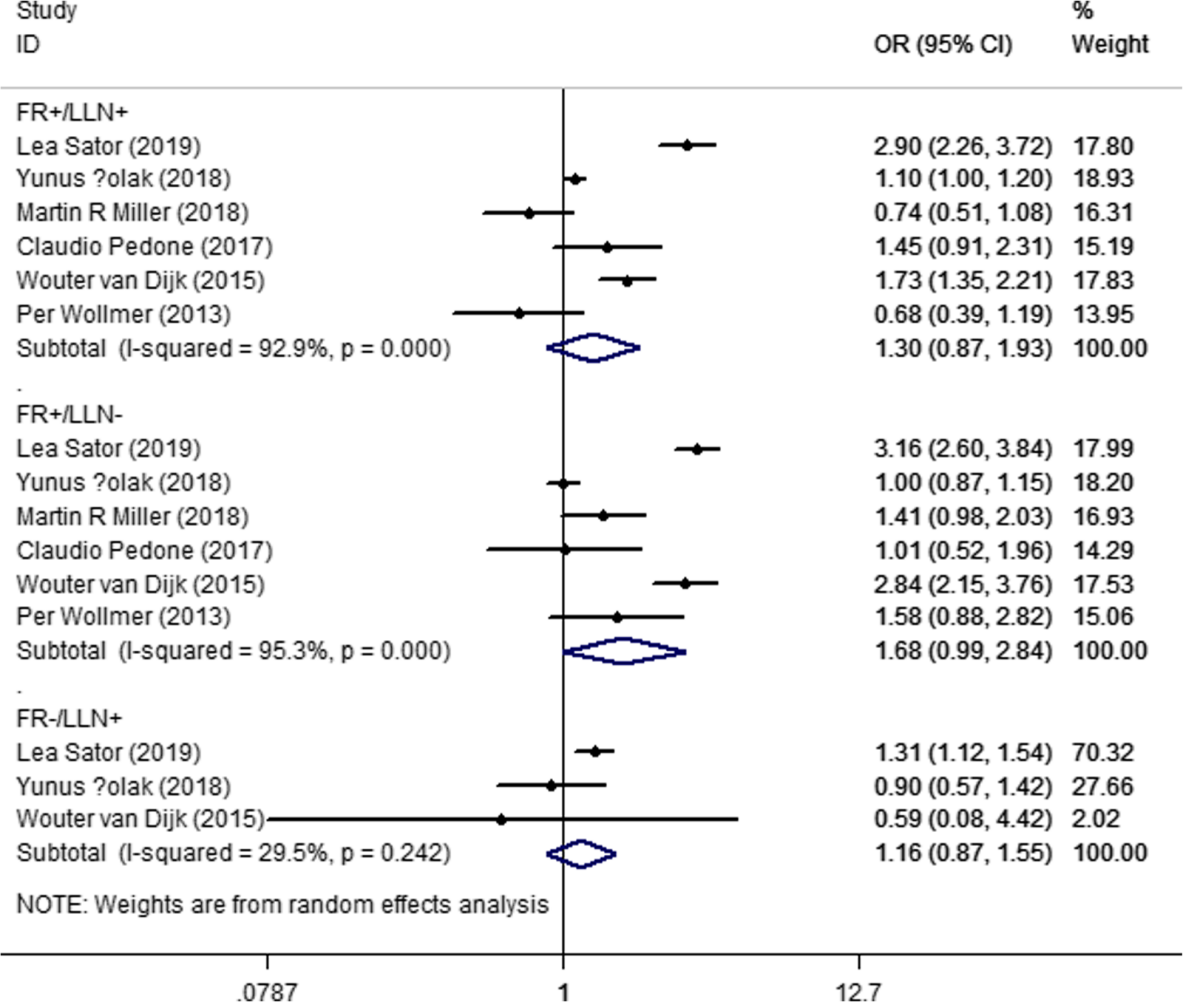
Forest plot of heart disease for different diagnostic criteria, compared with those met neither (FR+/LLN+: FEV1/FEV < 0.7 and FEV1/FVC < LLN, FR+/LLN-: FEV1/FEV < 0.7 but FEV1/FVC > LLN, FR−/LLN+: FEV1/FEV ≥ 0.7 but FEV1/FVC < LLN)

The comorbidities consisted of heart disease, heart failure, stroke, and diabetes. Six studies reported patients with heart disease. Our analysis found that when compared with patients who didn’t meet any criterion, there was no significant difference with those who met 1 or 2 criteria (OR _FR+/LLN+_ = 1.30, 95% CI = 0.87–1.93; OR _FR+/LLN-_ = 1.68, 95% CI = 0.99–2.84; OR _FR−/LLN+_ = 1.16, 95% CI = 0.87–1.55) (Fig. 4). The meta-analysis for heart failure, stroke, and diabetes showed no significant difference between the subjects or for the patients who fulfilled the LLN or FR criteria (Supplementary S3–5). The results of the sensitivity analysis did not change after excluding the studies one by one.

## Discussion

This systematic review and meta-analysis compared different studies for the risk of death, exacerbation of COPD, and comorbidities. There were several key findings: patients who met any 1 of the criterion for COPD were more likely to die than those met neither, individuals with airflow limitation according to FR were more likely to be worse according to symptoms. Irrespective of whether the participants met any criterion or not, the risk of developing heart disease, heart failure, stroke, and diabetes was not significantly different between the participants.

According to LLN or FR criteria, patients with airflow limitation had a higher risk of all-cause mortality; and according to FR, individuals with airflow limitation were more likely to be worse, which concurs with previous studies [24–26, 31]. Mannino DM et al. demonstrated a 4-fold risk of all-cause mortality in patients who met the criteria for LLN over an 18-year follow-up [25]; and in the same cohort, Akkermans RP et al. demonstrated that patients could suffer exacerbation at the first follow-up year according to the FR criterion [31]. Researchers also reported that patients with airflow limitation according to FR may account for poorer prognosis, including a higher risk of hospitalization and premature mortality [32]. One possible reason for such a phenomenon is the existence of airway hyper-responsiveness. Previous studies have shown that airway hyper-responsiveness may be an independent risk factor for mortality and an indicator of lung function decline [31, 33, 34]. Another possible explanation is that, as gas retention may occur in the early stages of COPD, the patient is worsened, and total lung capacity increases.

The results of mortality and exacerbation had high heterogeneity. There are several possible reasons for this: First, the severity of the participants included in the original studies was different. There was a close relationship between the level of severity and the risk of mortality and exacerbation. This meta-analysis demonstrated that participants with moderate airflow limitation may be more frequently hospitalized [35]. The risk of exacerbation was more frequent in the severe and very severe COPD patients [36]. Second, the proportion of ever-smoker and current-smoker varied; with the highest proportion at 84.5%. Previous studies have reported that the prevalence of COPD increased in smokers [37]; and that they have higher rates of lung function abnormalities and mortality [38]. Evidence has shown that smoking is one of the risk factors for COPD and that it can increase the lung’s total burden of inhaled particles and gases which in turn worsens a patient’s condition [36, 39–42].. Third, the process of data extraction might also be a source of heterogeneity. In this meta-analysis, some data were not available in the original studies and needed to be extracted by software. This might have caused some errors. Finally, the use of different spirometers and the different experience level of the clinicians could also have been sources for heterogeneity.

According to either the LLN or FR criteria, individuals with airflow limitations seem to have no significant difference for the risk of developing co-morbidities such as heart disease, stroke, heart failure, and diabetes mellitus, which is discordant with previous studies. Studies have demonstrated that the morbidities of COPD are affected by cardiovascular disease, diabetes mellitus, etc. [37, 43]. These chronic conditions are very closely related to age and sex. Although it is still unclear whether healthy aging will lead to COPD, or whether age reflects the sum of cumulative exposures throughout life, it is clear that age is a risk factor for COPD [44]. For a long time heart failure was considered to be closely related to airflow limitation and it was a challenge for doctors to diagnose patients with respiratory disease [45–47]. Yunus Çolak et al. demonstrated that heart failure could mimic airflow limitation in patients with COPD [21]. Therefore, it is doubtful whether clinicians should suspect cardiovascular disease in patients with airflow limitation according to FR only. A previous study also reported a poorer cardiopulmonary function in the LLN+/FR- group and no difference in the LLN−/FR+ group as compared to the control group [48]. Comorbidities can independently influence mortality and hospitalization in patients with COPD [49], so they deserve specific attention.

Heterogeneity was also high for the comorbidities. One possible reason for this is that the proportion of males in the original studies was unbalanced; the proportion of males varied from 23.1 to 100%. Another is the uneven age distribution of the population included in the original studies. The ages of the included participants in the original studies are different. The youngest and the oldest participants were 20 years and 100 years of age respectively. Several studies have demonstrated that the prevalence of COPD increased steeply with increasing age, especially among those > 60 years of age [37, 43, 50, 51]. Therefore, the studies which included participants > 60 years of age may have caused the heterogeneity.

This meta-analysis has several limitations. On one hand, because of the limited data in the original studies, we could not avoid the information bias. Several data for OR were calculated by the Revman software, and some mortality data for HR were extracted from the Kaplan Meier curve (K-M curve), which might have made our results less accurate. On the other hand, although only a few studies have described the prevalence of COPD for patients < 40 years of age, some studies have shown that patients < 40 years of age with COPD still account for a large portion of the total [4, 52]. Recognizing the characteristics and prognosis in COPD patients < 40 years of age is necessary. Therefore, a large number of studies are needed in the future to explore this group of patients. This is the only way to understand the overall disease characteristics of COPD and corresponding, take measures to reduce the burden of COPD and its effects on our economy and society.

## Conclusions

Patients who meet any one of the 2 diagnostic criteria are more likely to have higher mortality as compared to those who meet neither criterion; those who meet only FR are more likely to exacerbate. Considering the influence of developing co-morbidities, we found that in patients who met only the LLN criteria, the risk for developing heart disease, stroke, heart failure, and diabetes mellitus were not different from those meet neither criterion.

## Supplementary information

**Additional file 1 : Appendix S1**. Search strategies in databases. Supplementary S1: Quality assess for cohort studies (NOS). Supplementary S2: Quality assess for cross-sectional studies (AHRQ). Supplementary S3: Forest plot of heart failure for different diagnostic criteria, compared with those met neither). Supplementary S4: Forest plot of stroke for different diagnostic criteria, compared with those met neither. Supplementary S5: Forest plot of diabetes mellitus for different diagnostic criteria, compared with those met neither.

## Data Availability

All data generated or analyzed during this study are included in this published article.

## Abbreviations

AHRQ: the Agency for Healthcare Research and Quality
AMSTAR: Assessing the methodological quality of systematic reviews
ATS: American Thoracic Society
COPD: Chronic obstructive pulmonary disease
ERS: European Respiratory Society
FEV1: Forced expiratory volume in 1 s
FR: Fixed-ratio
FVC: Forced vital capacity
LLN: Lower limit of normal
NOS: Newcastle Ottawa Scale
PRISMA: Preferred reporting items for systematic reviews and meta-analysis
