# Supplementary material for: Assessment of comorbidities and prognosis in patients with COPD diagnosed with the fixed ratio and the lower limit of normal: a systematic review and meta-analysis
Source: Respir Res. 2020 Jul 16;21:189. doi: 10.1186/s12931-020-01450-9 (PMC7364614; doi:10.1186/s12931-020-01450-9)
Supplement: Supplementary file 1 — Additional file 1 : Appendix S1. Search strategies in databases. Supplementary S1: Quality assess for cohort studies (NOS). Supplementary S2: Quality assess for cross-sectional studies (AHRQ). Supplementary S3: Forest plot of heart failure for different diagnostic criteria, compared with those met neither). Supplementary S4: Forest plot of stroke for different diagnostic criteria, compared with those met neither. Supplementary S5: Forest plot of diabetes mellitus for different diagnostic criteria, compared with those met neither. [file 12931_2020_1450_MOESM1_ESM.doc]

Supplementary Materia

**Appendix S1: Search strategies in databases**

**Search strategy in PubMed:**

((((((((((((((("Pulmonary Disease, Chronic Obstructive"[Mesh]) OR (((((((((COPD[Title/Abstract]) OR Chronic Obstructive Pulmonary Disease[Title/Abstract]) OR COAD[Title/Abstract]) OR Chronic Obstructive Airway Disease[Title/Abstract]) OR Chronic Obstructive Lung Disease[Title/Abstract]) OR Airflow Obstruction, Chronic[Title/Abstract]) OR Airflow Obstructions, Chronic[Title/Abstract]) OR Chronic Airflow Obstructions[Title/Abstract]) OR Chronic Airflow Obstruction[Title/Abstract])) OR "Lung Diseases, Obstructive"[Mesh]) OR (((((((Lung Disease, Obstructive[Title/Abstract]) OR Obstructive Lung Disease[Title/Abstract]) OR Obstructive Lung Diseases[Title/Abstract]) OR Obstructive Pulmonary Diseases[Title/Abstract]) OR Obstructive Pulmonary Disease[Title/Abstract]) OR Pulmonary Disease, Obstructive[Title/Abstract]) OR Pulmonary Diseases, Obstructive[Title/Abstract])) OR ((Bronchitis, Chronic[Title/Abstract]) OR Chronic Bronchitis[Title/Abstract])) OR "Pulmonary Emphysema"[Mesh]) OR (((((((((((((((((((((((Emphysemas, Pulmonary[Title/Abstract]) OR Pulmonary Emphysemas[Title/Abstract]) OR Emphysema, Pulmonary[Title/Abstract]) OR Focal Emphysema[Title/Abstract]) OR Emphysema, Focal[Title/Abstract]) OR Emphysemas, Focal[Title/Abstract]) OR Focal Emphysemas[Title/Abstract]) OR Panacinar Emphysema[Title/Abstract]) OR Emphysema, Panacinar[Title/Abstract]) OR Emphysemas, Panacinar[Title/Abstract]) OR Panacinar Emphysemas[Title/Abstract]) OR Panlobular Emphysema[Title/Abstract]) OR Emphysema, Panlobular[Title/Abstract]) OR Emphysemas, Panlobular[Title/Abstract]) OR Panlobular Emphysemas[Title/Abstract]) OR Centriacinar Emphysema[Title/Abstract]) OR Centriacinar Emphysemas[Title/Abstract]) OR Emphysema, Centriacinar[Title/Abstract]) OR Emphysemas, Centriacinar[Title/Abstract]) OR Centrilobular Emphysema[Title/Abstract]) OR Centrilobular Emphysemas[Title/Abstract]) OR Emphysema, Centrilobular[Title/Abstract]) OR Emphysemas, Centrilobular[Title/Abstract])))))))))) AND (((((fixed ratio[Title/Abstract]) OR fixed-ratio[Title/Abstract]) OR FEV1/FVC＜0.7[Title/Abstract])) OR ((LLN[Title/Abstract]) OR Lower limit of normal[Title/Abstract]))

**Search strategy in Embase**

Session Results

.......................................................

No. Query Results

#14. #10 AND #13

#13. #11 OR #12

#12. 'lower limit of normal':ab,ti OR 'LLN':ab,ti

#11. 'fixed ratio'/ OR 'fixed-ratio':ab,ti OR 'FEV1/FVC＜0.7':ab,ti

#10. #5 OR #7 OR #9

#9. #3 OR #8

#8. 'bronchitis chronica':ab,ti OR 'bronchitis, chronic':ab,ti OR 'chronic bronchus

infection':ab,ti

#7. #2 OR #6

#6. 'bulbous emphysema':ab,ti OR 'bullous emphysema':ab,ti OR 'centrilobular emphysema':ab,ti OR 'chronic lung emphysema':ab,ti OR 'emphysema pulmonale':ab,ti OR 'emphysema, bullous':ab,ti OR 'intrapulmonary interstitial emphysema':ab,ti OR 'lobular emphysema':ab,ti OR 'lung bullous emphysema':ab,ti OR 'lung interstitial emphysema':ab,ti OR 'panacinar emphysema':ab,ti OR 'pneumatosis pulmonum':ab,ti OR 'pneumonectasia':ab,ti OR 'pulmonary emphysema':ab,ti OR 'unilateral pulmonary emphysema':ab,ti OR 'volumen pulmonum auctum':ab,ti

#5. #1 OR #4

#4. 'chronic airflow obstruction':ab,ti OR 'chronic airway obstruction':ab,ti OR 'chronic obstructive bronchitis':ab,ti OR 'chronic obstructive bronchopulmonary disease':ab,ti OR 'chronic obstructive lung disorder':ab,ti OR 'chronic obstructive pulmonary disease':ab,ti OR 'chronic obstructive pulmonary disorder':ab,ti OR 'chronic obstructive respiratory disease':ab,ti OR 'copd':ab,ti OR 'lung chronic obstructive disease':ab,ti OR 'lung disease, chronic obstructive':ab,ti OR 'lung diseases, obstructive':ab,ti OR 'obstructive lung disease':ab,ti OR 'obstructive lung disease, chronic':ab,ti OR 'obstructive pulmonary disease':ab,ti OR 'obstructive respiratory disease':ab,ti OR 'obstructive respiratory tract disease':ab,ti OR 'pulmonary disease, chronic obstructive':ab,ti OR 'pulmonary disorder, chronic obstructive':ab,ti

#3. 'chronic bronchitis'/exp

#2. 'lung emphysema'/exp

#1. 'chronic obstructive lung disease'/exp

.......................................................

**Search strategy in Cochrane Library**

ID Search

#1 MeSH descriptor: [Lung Diseases, Obstructive] explode all trees

#2 Pulmonary Disease, Obstructive

#3 Obstructive Pulmonary Diseases

#4 Obstructive Lung Diseases

#5 Obstructive Lung Disease

#6 Lung Disease, Obstructive

#7 Pulmonary Diseases, Obstructive

#8 Obstructive Pulmonary Disease

#9 #1 or #2 or #3 or #4 or #5 or #6 or #7 or #8

#10 MeSH descriptor: [Bronchitis, Chronic] explode all trees

#11 Chronic Bronchitis

#12 #10 or #11

#13 MeSH descriptor: [Pulmonary Emphysema] explode all trees

#14 Emphysema, Centrilobular

#15 Centrilobular Emphysemas

#16 Centrilobular Emphysema

#17 Centriacinar Emphysema

#18 Emphysema, Centriacinar

#19 Emphysemas, Centriacinar

#20 Centriacinar Emphysemas

#21 Emphysemas, Centrilobular

#22 Emphysemas, Pulmonary

#23 Emphysema, Pulmonary

#24 Pulmonary Emphysemas

#25 Panlobular Emphysemas

#26 Panacinar Emphysema

#27 Emphysemas, Panacinar

#28 Emphysema, Panlobular

#29 Panlobular Emphysema

#30 Panacinar Emphysemas

#31 Emphysemas, Panlobular

#32 Emphysema, Panacinar

#33 Emphysemas, Focal

#34 Focal Emphysema

#35 Focal Emphysemas

#36 Emphysema, Focal

#37 #13 or #14 or #15 or #16 or #17 or #18 or #19 or #20 or #21 or #22 or #23 or #24 or #25 or #26 or 27 or #28 or #29 or #30 or #31 or #32 or #33 or #34 or #35 or #36

#38 MeSH descriptor: [Pulmonary Disease, Chronic Obstructive] explode all trees

#39 Chronic Obstructive Lung Disease

#40 Chronic Obstructive Airway Disease

#41 COPD

#42 Chronic Obstructive Pulmonary Disease

#43 COAD

#44 Airflow Obstructions, Chronic

#45 Airflow Obstruction, Chronic

#46 Chronic Airflow Obstruction

#47 Chronic Airflow Obstructions

#48 #38 or #39 or #40 or #41 or #42 or #43 or #44 or #45 or #46 or #47

#49 #9 or #12 or #37 or #48

#50 fixed-ratio

#51 fixed ratio

#52 FEV1/FVC＜0.7

#53 #50 or #51 or #52

#54 lower limit of normal

#55 LLN

#56 #54 or #55

#57 #53 or #56

#58 #49 and #57

**Search strategy in Web of Science**

#01 Theme: (fixed-ratio) OR Theme: (fixed ratio) OR Theme: (FEV1/FVC＜0.7) OR Theme: (lower limit of normal) OR Theme: (LLN)

#02 Theme:  (Pulmonary Disease, Chronic Obstructive) OR Theme: (COPD) OR Theme: (Chronic Obstructive Pulmonary Disease) OR Theme: (COAD) OR Theme: (Chronic Obstructive Airway Disease) OR Theme: (Chronic Obstructive Lung Disease) OR Theme: (Airflow Obstruction, Chronic) OR Theme: (Airflow Obstructions, Chronic) ORTheme: (Chronic Airflow Obstructions) OR Theme: (Chronic Airflow Obstruction)

#03 Theme: (Lung Diseases, Obstructive) OR Theme: (Lung Disease, Obstructive) OR Theme: (Obstructive Lung Disease) OR Theme: (Obstructive Lung Diseases) OR Theme: (Obstructive Pulmonary Diseases) OR Theme: (Obstructive Pulmonary Disease) OR Theme: (Pulmonary Disease, Obstructive) OR Theme: (Pulmonary Diseases, Obstructive)

#04 Theme: (Bronchitis, Chronic) OR Theme: (Chronic Bronchitis)

#05 (((((((((((((((((((((((Theme: (Pulmonary Emphysema) OR Theme: (emphysema, Pulmonary)) OR Theme: (Pulmonary emphysema)) OR Theme: (Emphysema, Pulmonary)) OR Theme: (Focal Emphysema)) OR Theme: (Emphysema, Focal)) OR Theme: (emphysema, Focal)) OR Theme: (Focal emphysema)) OR Theme: (Panacinar Emphysema)) OR Theme: (Emphysema, Panacinar)) OR Theme: (emphysema, Panacinar)) OR Theme: (Panacinar emphysema)) OR Theme: (panlobar Emphysema)) OR Theme: (Emphysema, panlobar)) OR Theme: (emphysema, panlobar)) OR Theme: (panlobar emphysema)) OR Theme: (centroacinar Emphysema)) OR Theme: (centroacinar emphysema)) OR Theme: (Emphysema, centroacinar)) OR Theme: (emphysema, centroacinar)) OR Theme: (Centrilobular Emphysema)) OR Theme: (Centrilobular emphysema)) OR Theme: (Emphysema, Centrilobular)) OR Theme: (emphysema, Centrilobular))

#06 #5 OR #4 OR #3 OR #2

#07 #6 AND #1

**Supplementary S1: Quality assess for cohort studies (NOS)**

| Inclusion studies | | Selection | | | | Comparability | Outcome | | | Score |
| --- | --- | --- | --- | --- | --- | --- | --- | --- | --- | --- |
| Study | Year | ① | ② | ③ | ④ | ⑤ | ⑥ | ⑦ | ⑧ | - |
| Lea Sator | 2019 | 1 | 1 | 1 | 0 | 1 | 1 | 1 | 0 | 6 |
| Yunus Çolak | 2018 | 1 | 1 | 1 | 1 | 1 | 1 | 1 | 1 | 8 |
| Claudio Pedone | 2017 | 1 | 1 | 1 | 1 | 1 | 1 | 1 | 1 | 8 |
| Suneela Zaigham | 2015 | 1 | 1 | 1 | 1 | 1 | 1 | 1 | 1 | 8 |
| Eralda Turkeshi | 2015 | 1 | 1 | 1 | 1 | 1 | 1 | 1 | 1 | 8 |
| Surya P Bhatt | 2014 | 1 | 1 | 1 | 1 | 1 | 1 | 1 | 1 | 8 |
| Per Wollmer | 2013 | 1 | 1 | 1 | 0 | 1 | 1 | 1 | 1 | 7 |
| Firdaus A | 2013 | 1 | 1 | 1 | 0 | 1 | 1 | 1 | 0 | 6 |
| David M Mannino | 2006 | 1 | 1 | 1 | 0 | 1 | 1 | 1 | 1 | 7 |
| David M. Mannino | 2012 | 1 | 1 | 1 | 0 | 1 | 1 | 1 | 1 | 7 |
| Reinier P. Akkermans | 2012 | 1 | 1 | 1 | 1 | 1 | 1 | 1 | 1 | 8 |

Abbreviations: ①. Representativeness of the exposed cohort, ②. Selection of the non exposed cohort, ③. Ascertainment of exposure, ④.Demonstration that outcome of interest was not present at start of study, ⑤.Comparability of cohorts on the basis of the design or analysis, ⑥.Assessment of outcome, ⑦.Was follow-up long enough for outcomes to occur, ⑧.Adequacy of follow up of cohorts

**Supplementary S2: Quality assess for cross-sectional studies (AHRQ)**

| Study | Year | ① | ② | ③ | ④ | ⑤ | ⑥ | ⑦ | ⑧ | ⑨ | ⑩ | ⑪ | Score |
| --- | --- | --- | --- | --- | --- | --- | --- | --- | --- | --- | --- | --- | --- |
| Martin R Miller | 2018 | 1 | 1 | 1 | 1 | 1 | 1 | 1 | 1 | 0 | 1 | 1 | 10 |
| Wouter van Dijk | 2015 | 1 | 1 | 1 | 1 | 0 | 1 | 1 | 1 | 0 | 1 | 0 | 8 |

Abbreviations: ①. Define the source of imformation (survey, record review), ②. List inclusion and exclusion criteria for exposed and unexposed subjects (cases and control ) or refer to previous publications, ③. Indicate time period used for identifying patients, ④. Indicate whether or not subjects were consecutive if not population-based, ⑤. Indicate if evaluators of subjective components of study were masked to other aspects of the status of the participants, ⑥. Describe any assessmets undertaken for quality assurance purposes (eg.test/retest of primary outcome measurements ), ⑦. Explain any patient exclusions from anaysis, ⑧. Describe how confounding was assessed and/or controlled; ⑨. If applicable,explain how missing data were handled in the analysis; ⑩. Summarize patient response rates and completeness of data collection; ⑪. Clarify what follow-up, if any, was expected and the percentage of patients for which incomplete data or follow-up was obtained.


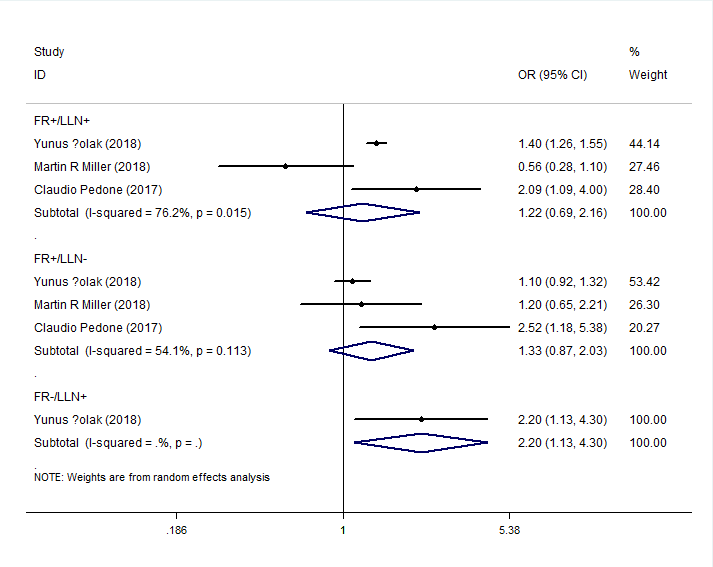


Supplementary S3: Forest plot of heart failure for different diagnostic criteria, compared with those met neither.


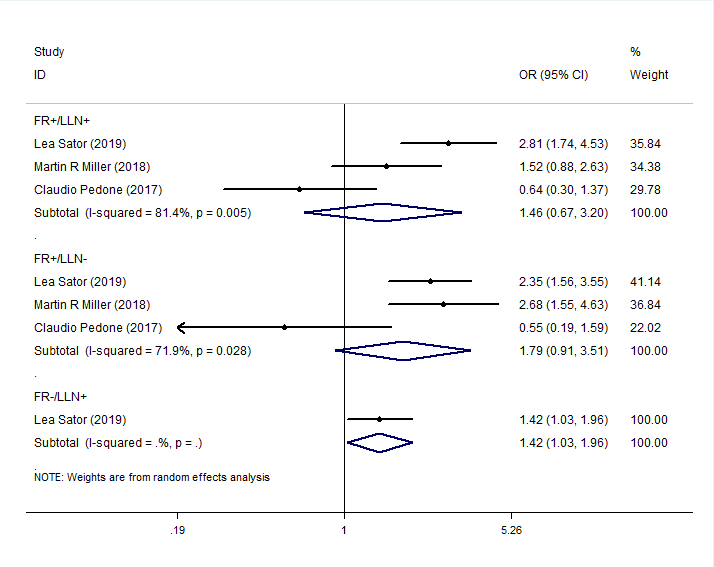


Supplementary S4: Forest plot of stroke for different diagnostic criteria, compared with those met neither.


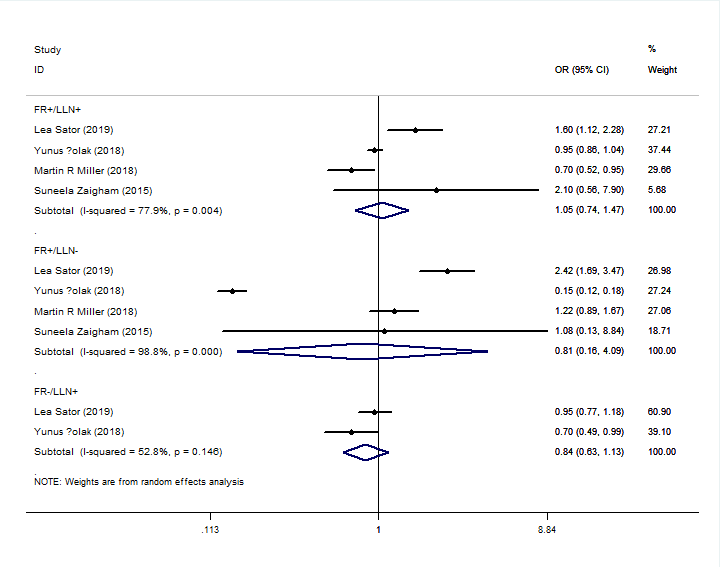


Supplementary S5: Forest plot of diabetes mellitus for different diagnostic criteria, compared with those met neither.
